# Survival trend in metastatic prostate cancer two decades of real-world data on overall survival from Germany

**DOI:** 10.1038/s41391-026-01092-w

**Published:** 2026-02-24

**Authors:** Marcus Sondermann, Anton Stehr, Christopher Hirtsiefer, Viktoria Menzel, Nina Buttmann-Schweiger, Paul Wilhelm Flemming, Klaus Kraywinkel, Christian Thomas, Katharina Boehm

**Affiliations:** 1 Universitätsklinikum Carl-Gustav-Carus Dresden, Department of Urology, Technische Universität Dresden, Dresden, Germany; 2 Department of Epidemiology and Health Monitoring, German Centre for Cancer Registry Data (ZfKD), Berlin, Germany; 3 Vancouver Prostate Centre, Department of Urologic Sciences, Medical Faculty, University of British Columbia, Vancouver, Canada

**Keywords:** Outcomes research, Translational research

## Abstract

**Background:**

The management of metastatic prostate cancer (mPCa) has undergone revolutionary changes over the past two decades with the introduction of novel hormonal agents, chemotherapy combinations, PARP inhibitors, and radioligand therapies. This study evaluates the real-world impact of these therapeutic advances on overall survival (OS) in Germany.

**Methods:**

We analyzed data from the German national cancer registry covering 657,499 prostate cancer cases diagnosed between 1999 and 2021. After exclusions, 54,890 patients with de novo metastatic disease (M1) were included. Primary outcome was median OS. Secondary outcomes included 3-, 5-, and 10-year survival rates. Time series analysis assessed temporal trends using augmented Dickey-Fuller tests and joinpoint regression.

**Results:**

Median OS for M1 patients improved from 31.0 months (95% CI: 29.8–32.2) in 1999 to 37.0 months (35.6–38.4) in 2019 (*p* < 0.001). This 19.4% improvement exceeded general life expectancy gains. Age-stratified analysis revealed disparate benefits: patients < 70 years experienced improvement from 34.0 to 49.0 months ( + 44.1%), while those ≥ 70 years showed minimal change (28.0 to 29.0 months, +3.6%). Three-year survival increased from 45.1% to 50.9% (*p* = 0.004), with younger patients achieving 61.3% versus 44.0% for older patients by 2019. Multivariate Cox regression confirmed diagnosis year as an independent predictor (HR 0.96, 95% CI: 0.96–0.97, *p* < 0.001).

**Conclusions:**

Real-world data confirm meaningful survival improvements in metastatic prostate cancer over two decades, validating the translation of clinical trial efficacy into routine practice. However, the pronounced age-related disparity suggests potential undertreatment of elderly patients and highlights the need for age-adapted treatment strategies.

**Figure:**
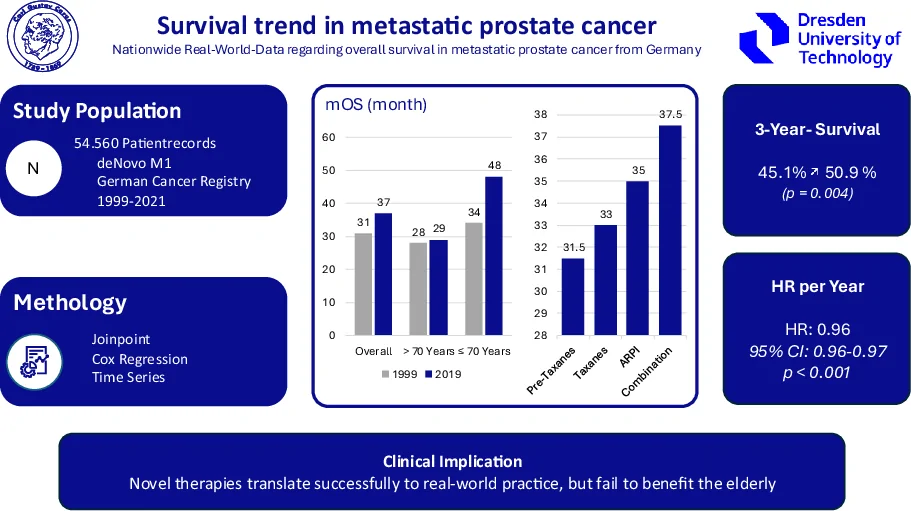

## Introduction

Prostate cancer remains the most prevalent malignancy among men in Western populations, with metastatic disease representing a critical therapeutic challenge despite recent advances [1]. The therapeutic landscape for metastatic prostate cancer (mPCa) has undergone unprecedented transformation over the past two decades, evolving from simple androgen deprivation therapy (ADT) to complex multimodal approaches incorporating novel hormonal agents, chemotherapy, targeted therapies, and radioligand treatments [2].

The introduction of docetaxel chemotherapy in 2004 marked the first breakthrough in improving survival for metastatic castration-resistant prostate cancer (mCRPC), demonstrating a significant 2.4-month survival benefit [3]. Subsequently, the therapeutic armamentarium expanded rapidly with second-generation antiandrogens including abiraterone in 2011 [4], enzalutamide in 2014 [5, 6], apalutamide in 2018 [7], and darolutamide in 2019 [8], each demonstrating survival benefits in both hormone-sensitive and castration-resistant settings.

The CHAARTED and STAMPEDE trials revolutionized treatment paradigms by establishing early chemotherapy combined with ADT for high-volume metastatic hormone-sensitive disease, achieving median overall survival exceeding 57 months [9, 10]. The LATITUDE trial further validated early intensification with abiraterone plus ADT, demonstrating a 16.8-month survival advantage in high-risk disease [11].

More recently, precision medicine approaches have emerged with PARP inhibitors for patients harboring homologous recombination repair deficiencies [12], and ^177^Lu-PSMA- radioligand therapy has shown remarkable efficacy in heavily pretreated mCRPC [13]. The VISION trial demonstrated a 4-month overall survival benefit with ^177^Lu-PSMA, while triple therapy combining ADT, docetaxel, and darolutamide in the ARASENS trial has pushed median survival boundaries beyond 48 months for de novo metastatic disease [13, 14].

Despite these therapeutic advances demonstrated in clinical trials, their real-world impact on population-level outcomes remains incompletely characterized. Clinical trial populations often underrepresent elderly patients and those with comorbidities, potentially limiting generalizability [15]. Furthermore, the translation of trial efficacy into routine clinical practice depends on factors including healthcare access, physician adoption, and patient selection [16, 17].

This study leverages comprehensive German cancer registry data spanning 1999-2021 to evaluate temporal trends in overall survival for patients with de novo metastatic prostate cancer, assessing whether clinical trial advances have translated into meaningful population-level improvements.

## Methods

### Data source and patient selection

We utilized data from the German Centre for Cancer Registry Data (Zentrum für Krebsregisterdaten, ZfKD), which aggregates population-based cancer registrations from all 16 German federal states [18]. The dataset encompasses all prostate cancer diagnoses (ICD-10: C 61) registered between January 1, 1999, and December 31, 2021, with vital status follow-up through December 31, 2023. Data collection followed standardized protocols established by the Federal Cancer Registry Act [19] of 2009, with annual quality monitoring ensuring completeness exceeding 95% for mortality linkage.

From an initial cohort of 657,499 prostate cancer cases, we applied sequential exclusion criteria: autopsy-only diagnoses (*n* = 886), secondary malignancies (*n* = 114,068), death within 30 days of diagnosis (*n* = 10,589), age <30 years (*n* = 108), and missing M-stage information (*n* = 466,223). Of the remaining 531,848 patients, 342,437 had documented M0 disease, and 134,521 had missing or unknown M-stage (Mx). Patients with Mx status were classified as M0 based on clinical practice patterns. The final analytical cohort comprised 54,890 patients with de novo M1 disease.

### Statistical analysis

The primary endpoint was median overall survival (mOS), defined as time from initial diagnosis to death from any cause. Secondary endpoints included three- and five-year survival rates. Survival analyses employed Kaplan-Meier methodology with log-rank testing for group comparisons. Patients were stratified by age at diagnosis (Old: < 70 years vs. ≥ 70).

Time series analyses assessed temporal trends using augmented Dickey-Fuller (ADF) tests for stationarity and approximate difference-sign tests (DS) or Cox-Stuart-Test (CS) for monotonic trends [20, 21]. Joinpoint regression identified potential inflection points in survival trends, with permutation testing for model selection [22]. Details are descripted in supplementary methods.

Multivariate Cox proportional hazards regression evaluated independent predictors of survival, including diagnosis year, age, T-stage, N-stage, and UICC stage. Proportional hazards assumptions were tested using Schoenfeld residuals [23]. For variables violating proportionality assumptions, we employed stratified Cox models. Statistical significance was defined as *p* < 0.05. Analyses were performed using R version 4.3.0 with packages survival, survminer, and fable [24–28].

## Results

### Patient characteristics

Among 54,890 patients with de novo metastatic disease, median age at diagnosis was 72 years (IQR: 65–78). The annual incidence of M1 disease increased from 1,287 cases in 1999 to 3142 cases in 2019, partially reflecting improved detection and registry completeness. Baseline characteristics demonstrated expected distributions: T4 disease in 21%, N1 disease in 45%, and 75% mortality during follow-up of 20 years. Complete TNM staging was available for 67.8% of patients, with missing data more common in earlier years. The complete baseline characteristics are listed in Supplementary Table 1.

### Overall survival trends

**Fig. 1:**
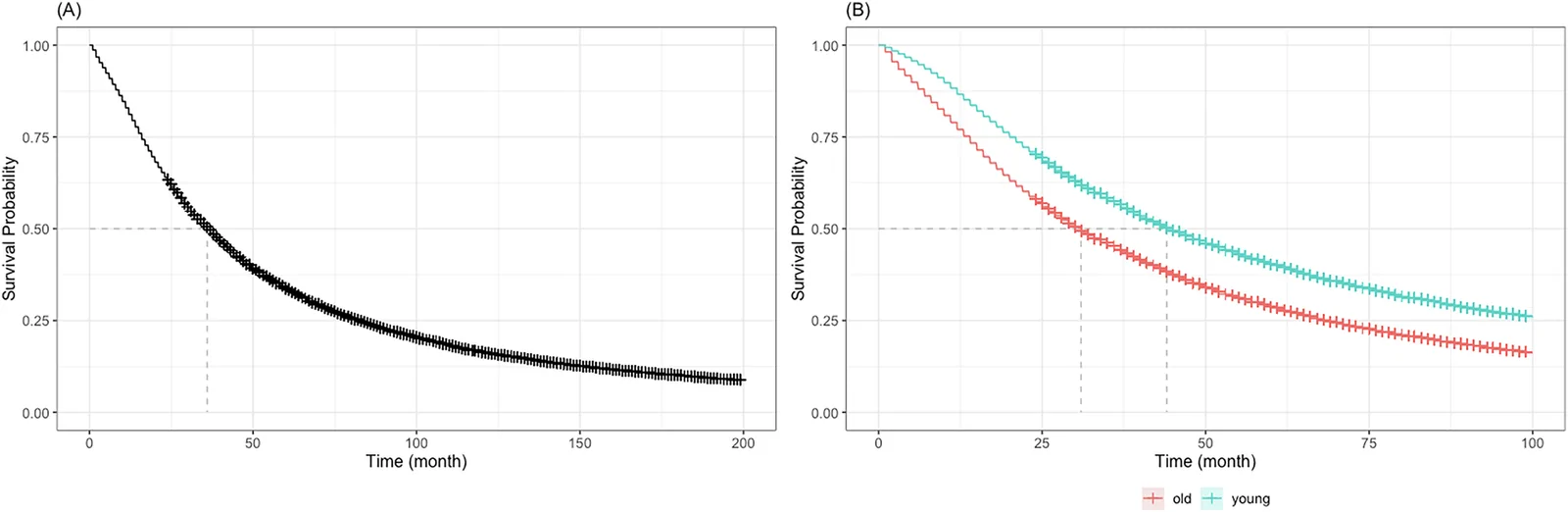
Overall survival in metastasis cohort. Kaplan-Meier Estimators for primary metastasized prostate cancer patient in our cohort. **A** Represents the overall cohort, as **B** Is divided by our defined age groups in old (red) and young (turquoise). Under each graph the risk table is placed.

**Fig. 2:**
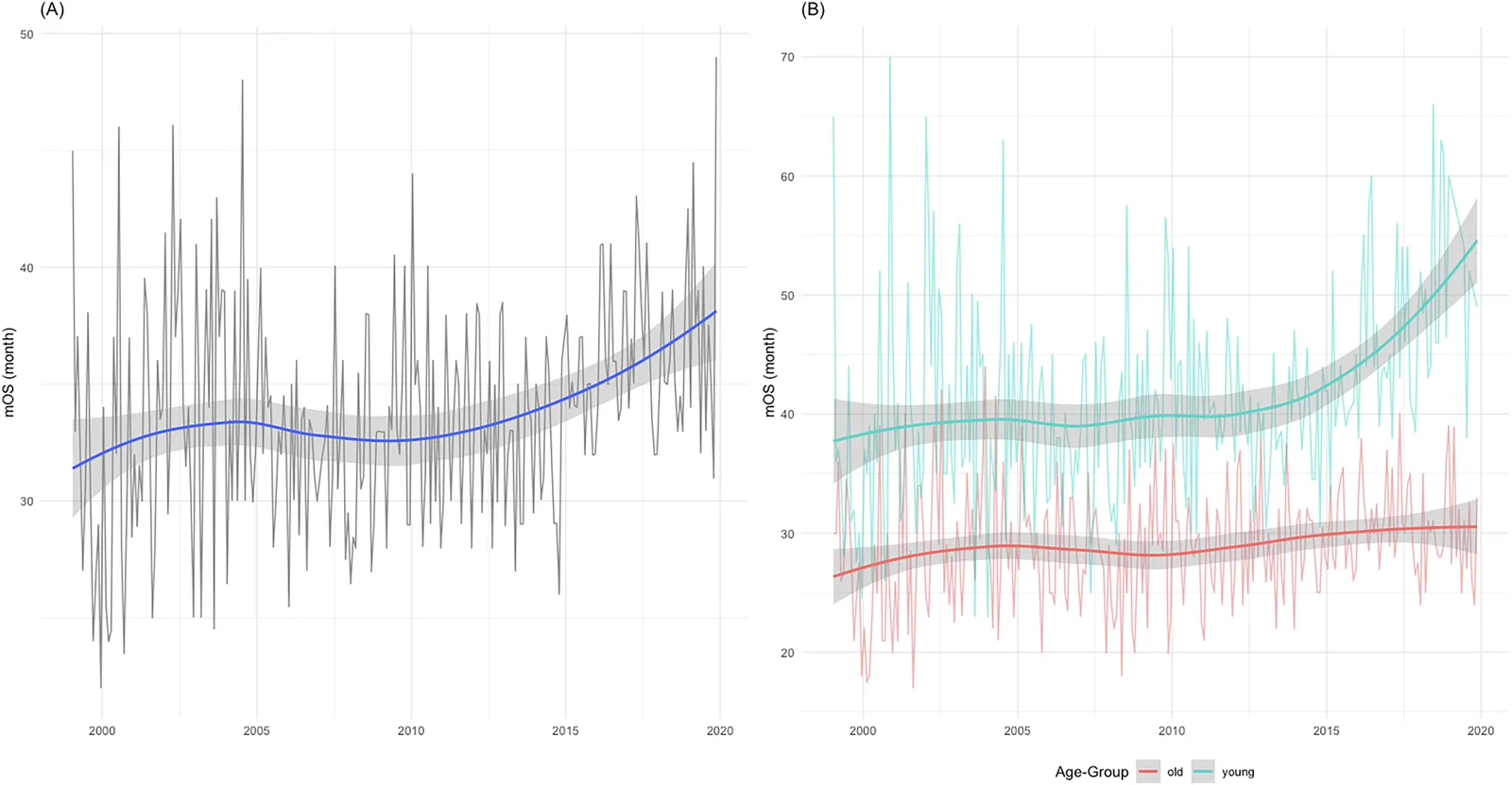
Median Overall Survial Rates. Overall **A** Median Overall Survival Time in month and splitted by age group **B**. In B the green line represents the younger patients as the red line represents the older patients. The grey overlay estimates the course over the years by a Gaussian model.

Median overall survival for the entire M1 cohort improved significantly from 31months (95% CI: 29.8–32.2) in 1999 to 37months (35.6–38.4) in 2019, representing a 19.4% relative improvement, as shown in Figs. 1, 2. Time series analysis confirmed non-stationary behavior (ADF: *p* = 0.831) with a significant positive trend (DS: *p* = 0.004). Linear regression demonstrated an average gain of 0.22 months with every year diagnosed later (95% CI: 0.12–0.32, *p* < 0.001).

Multivariate Cox regression identified diagnosis year as an independent predictor of improved survival (HR 0.96 per year, 95% CI: 0.96–0.97, *p* < 0.001), after adjustment for age, TNM-Stadium and UICC stage, with 99% power in tests. The proportional hazards assumption was violated (Grambsch-Therneau: *p* < 0.001), prompting stratified analysis that revealed differential temporal effects: the survival benefit was more pronounced in the early period ( ≤ median survival time, HR 0.89, 95% CI: 0.89–0.90) compared to late cohort of this study’s scope ( > median, HR 0.94, 95% CI: 0.94–0.95).

### Age-stratified outcomes

Profound age-related disparities emerged in survival improvements in metastatic disease. Patients <70 years experienced dramatic gains, with median OS increasing from 34 months in 1999 to 48.0 months in 2019 ( + 44.1%). In contrast, patients ≥ 70 years showed minimal improvement: 28 to 29 months ( + 3.6%). The age-stratified time series demonstrated non-stationary behavior for younger patients (ADF: *p* = 0.907) with significant positive trends (CS: *p* = 0.029), while older patients exhibited stationary patterns (ADF: *p* = 0.041) suggesting plateau effects.

Linear regression of annual survival data confirmed divergent trajectories: younger patients gained 0.44 months annually (95% CI: 0.28–0.60, *p* < 0.001), while older patients gained only 0.17 months annually (95% CI: 0.05–0.29, *p* = 0.007). The interaction between age group and diagnosis year was statistically significant (*p* = 0.003), confirming differential temporal effects.

### Three- and five-year survival

Three-year survival rates improved from 45.1% (95% CI: 43.2–47.0) in 1999 to 50.9% (49.4–52.4) in 2019, with joinpoint regression confirming linear improvement without inflection points ( + 0.19% annually, *p* = 0.004). Age stratification revealed a larger increase in younger patients (ADF: p = 0.89, DF: *p* = 0.0004) with increase by 0.5% per year (R^2^ = 0,57, *p* < 0.005)

Five-year survival increased modestly from 29.6% (27.8–31.4) to 34.1% (32.7–35.5) between 1999 and 2018, though trends showed greater variability (DS: *p* = 0.786). Younger patients demonstrated robust improvements from 35% to 43%, while older patients plateaued around 28% (CS: *p* = 0.50). Ten-year survival analysis was limited by insufficient follow-up for recent cohorts but suggested no significant improvements (*p* = 0.412), with rates remaining stable at approximately 15% for younger and 8% for older patients. The apparent decline in 10-year survival rates in recent cohorts reflects insufficient follow-up time, not actual deterioration in outcomes. Patients diagnosed after 2013 have not reached documented 10-year follow-up by authorities, so their long-term survival cannot be reliably estimated.

### Survival by diagnostic era

To contextualize improvements with therapeutic milestones, we analyzed survival by treatment eras. The pre-taxanes era (1999–2003) demonstrated mOS of 31.5 months (30.8–32.2). The taxanes era (2004–2010) showed initial improvements to 33.0 months (32.5–33.5). The androgen receptor pathway inhibition (ARPI) era (2011–2016) achieved 35.0 months (34.4–35.6), while the contemporary combination therapy era (2017–2021) reached 37.5 months (36.8–38.2), though complete follow-up remains pending for recent cohorts. These results are shown in Table 1 and Supplementary Table 6.

**Table 1:** Treatment success by Treatment era.

Era	Years	*n*	Median OS (95% CI)	Comparison Test	*p*-value
Pre-Taxanes	1999-2003	6474	31.5 (30.8-32.2)	Reference	-
Taxanes	2004-2010	11,234	33.0 (32.5-33.5)	Log-rank vs. Pre-Taxanes	0.018
ARPI	2011-2016	17,823	35.0 (34.4-35.6)	Log-rank vs. Taxanes	0.002
Contemporary	2017-2021	19,359	37.5 (36.8-38.2)	Log-rank vs ARPI	<0.001

Subgroup analysis by baseline characteristics revealed consistent improvements across T-stages and nodal status, though patients with T4 disease showed greater absolute gains (24 to 32 months) compared to T1-2 disease (40 to 44 months). UICC stage IV patients demonstrated the most substantial improvements, likely reflecting the impact of systemic therapy advances.

## Discussion

This comprehensive population-based analysis provides compelling real-world evidence that therapeutic advances in metastatic prostate cancer have translated into meaningful survival improvements at the population level. The 6-month improvement in median overall survival over two decades, while modest in absolute terms, represents a 19.4% relative gain that exceeds concurrent improvements in general life expectancy (6% increase nationally) [29], with a slower increase over time [30]. These findings support the idea of the cumulative impact of sequential therapeutic innovations, from docetaxel chemotherapy through novel hormonal agents to contemporary combination strategies.

The observed survival improvements align with expected benefits from pivotal clinical trials, though population-level gains appear attenuated compared to trial outcomes. While trials like CHAARTED and LATITUDE reported median survival exceeding 57 months with combination therapy [9, 11], our real-world median of 37 months in 2019 reflects the heterogeneous patient population, variable treatment uptake, and competing mortality risks in routine practice. This efficacy-effectiveness gap is well-recognized in oncology and underscores the importance of real-world evidence in contextualizing trial results [31].

While the temporal correlation between survival improvements and drug approvals is suggestive of therapeutic benefit, several confounding factors warrant consideration. First, advances in supportive care, including improved management of treatment-related adverse events, better palliative care services, and enhanced monitoring protocols. Second, stage migration. The introduction sensitive diagnostic modalities, as molecular Imaging, enables detection of metastatic disease at earlier stages than previously possible [32]. In Germany, PSMA-PET became available from 2015 onwards, with broader clinical adoption around 2017-2018. Patients who would previously have been classified as M0 based on conventional imaging may now be identified as M1 with minimal disease burden, artificially improving survival statistics for the M1 cohort [33]. Regional variation in PET availability across German federal states may also contribute to heterogeneity in observed outcomes. Third, improvements in surgical and radiotherapeutic techniques for oligometastatic disease contribute to improved survival independent of systemic therapy advances.

The striking age-related disparity in survival improvements raises questions about treatment equity and optimization in elderly patients. Younger patients ( < 70 years) experienced a 44% improvement in median survival, approaching contemporary trial benchmarks, while elderly patients ( ≥ 70 years) showed minimal gains ( + 3.6%) despite representing 59% of the metastatic population in this study. This disparity likely reflects multifactorial influences including treatment selection bias, competing comorbidities, functional status limitations, and potentially inappropriate therapeutic nihilism in elderly patients [34].

Several factors may explain the limited improvements in elderly patients. First, clinical trial underrepresentation of elderly patients limits evidence-based treatment guidelines for this population [15]. Second, concerns about tolerability may lead to undertreatment, despite evidence supporting the safety of novel agents in fit elderly patients [35]. Third, competing mortality risks dilute cancer-specific survival benefits. Cause-specific mortality data were unavailable, limiting assessment of competing risks. This is particularly relevant for elderly patients ( ≥ 70 years), where non-cancer mortality significantly influences overall survival [36]. This may overestimate the degree of ‘undertreatment’ in elderly patients. The true cancer-specific survival benefit in elderly patients may be larger than overall survival figures suggest. It may also indicate appropriate treatment de-intensification in patients with limited life expectancy from other causes. Without cause-of-death data, we cannot distinguish between these interpretations. Although our findings align with registry studies from other countries showing similar age-related disparities [37].

The temporal patterns observed suggest accelerating improvements coinciding with specific therapeutic introductions. The periodic trends around 2011 corresponds with abiraterone and enzalutamide availability, while continued improvements through 2019 likely reflect early intensification strategies with combination therapy. The greater benefit observed in early versus late follow-up periods suggests that modern therapies primarily extend initial disease control rather than altering ultimate disease trajectory, consistent with patterns observed in other advanced malignancies [38].

Notably, the plateau in 5- and 10-year survival rates suggests that while modern therapies extend short-term survival, achieving long-term disease control remains elusive for most patients. Due to the differences and short follow-up period this question cannot be answered seriously. Although a ceiling effect highlights the need for novel therapeutic approaches targeting resistance mechanisms and disease biology rather than incremental improvements to existing strategies.

### Strengths and limitations

This study’s primary strength lies in its comprehensive population-based design, capturing real-world outcomes across an entire national healthcare system with universal coverage. The 20-year observation period enables robust trend analysis across multiple therapeutic eras, while the large sample size provides statistical power to detect modest but clinically meaningful changes. The German healthcare system’s universal coverage minimizes selection bias related to insurance status, enhancing generalizability.

However, several limitations merit consideration. First, treatment-specific data were unavailable, precluding direct attribution of survival improvements to specific therapies. Without knowledge of patient specific treatment regimes, the association between drug approvals and survival gains remains associative. This represents a fundamental limitation of registry-based studies lacking treatment data.

Second, incomplete staging information (16. 5% missing T-stage) may introduce selection bias, though sensitivity analyses excluding incomplete cases yielded similar results.

Third, lack of PSA data prevents risk stratification or assessment of stage migration effects from improved diagnostics, particularly PSMA-PET imaging which became available in Germany from 2017 onwards.

Fourth, cause-specific mortality data were unavailable, limiting assessment of competing risks particularly in elderly patients. Finally, the study period predates widespread adoption of PSMA-PET imaging and radioligand therapy, which may further improve outcomes.

Fifth, these results are only applicable to patients in the German health system, where most patients receive treatment with minimal impact of costs.

### Clinical implications and future directions

Our findings have important implications for clinical practice and health policy. The documented survival improvements support continued investment in novel therapeutic development. Further implication lac in detailed information on used therapeutics and death causes. However, the pronounced age-related disparities demand targeted interventions to optimize elderly patient care, including geriatric assessment integration, adapted treatment protocols, and shared decision-making frameworks that balance survival benefits against quality-of-life considerations [39].

Future research should focus on identifying barriers to optimal treatment in elderly patients, developing risk-stratification tools to guide treatment selection, and evaluating implementation strategies to ensure equitable access to advances. Registry studies incorporating treatment data would enable assessment of specific therapeutic contributions to survival gains. As the BKRG was changed to collect clinical data by 2020, future treatments can be analyzed to reduce this limitation in the future. This way newer therapies including PSMA-targeted radioligand therapy and triple combination approaches disseminate into practice, continued monitoring through registry studies will be essential to assess their real-world impact.

## Conclusions

This large-scale population analysis demonstrates that therapeutic advances in metastatic prostate cancer have yielded meaningful survival improvements in real-world practice, with median overall survival improving by 19.4% over two decades. However, these benefits are predominantly restricted to younger patients, with elderly patients experiencing minimal gains despite comprising the majority of the metastatic population. These findings highlight both the success of therapeutic innovation and the persistent challenge of ensuring equitable benefit across all patient populations. As the therapeutic landscape continues evolving with precision medicine approaches and novel combination strategies, focused efforts to optimize treatment selection and delivery for elderly patients will be essential to maximize population-level impact.

## Supplementary information

Supplementary Material

## Data Availability

Individual-level data cannot be shared due to privacy regulations. Aggregated data supporting the findings are available through official request to: German Centre for Cancer Registry Data (ZfKD). Further Details are listed in the Supplementary Methods.
