# Supplementary material for: Survival trend in metastatic prostate cancer two decades of real-world data on overall survival from Germany
Source: Prostate Cancer Prostatic Dis. 2026 Feb 24;29(4):820–5. doi: 10.1038/s41391-026-01092-w (PMC13623643; doi:10.1038/s41391-026-01092-w)
Supplement: Supplementary file 1 [file 41391_2026_1092_MOESM1_ESM.docx]

Supplementary Material

# Additional Tables and Graphics

**Figures:**

[Figure 1: Overall Survival in Metastasis Cohort](#_Toc212894422)

[Figure 2: Median Overall Survial Rates](#_Toc212894423)

[Supplementary Figure 1: Survival Rates in differents years](#_Toc212894426)

[Supplementary Figure 2: Schoenfeld Residuals Plot for Proportional Hazards Assessment](#_Toc212894427)

**Tables:**

[Table 1: Treatment success by Treatment era](#_Toc212894441)

[Supplementary Table 1: Patient Characteristics by M-Stadium](#_Toc221822926)

[Supplementary Table 2: Annual 3-Year and 5-Year Overall Survival Rates](#_Toc221822927)

[Supplementary Table 3: Complete Case Analysis - Comparison of Patient Characteristics](#_Toc221822928)

[Supplementary Table 4: Three Year Survival Trends by Federal State](#_Toc221822929)

[Supplementary Table 5: Age-Stratified Analysis](#_Toc221822930)

[Supplementary Table 6: Treatment Era Comparisons](#_Toc221822931)

## Supplementary Table 1: Patient Characteristics by M-Stadium

|  | N | Overall (%) N = 657,499*^1^* | 0 (%) N = 602,609*^1^* | 1 (%) N = 54,890*^1^* |
| --- | --- | --- | --- | --- |
| T-Stadium | 624,434 |  |  |  |
| 0 |  | 241 (<0.1%) | 194 (<0.1%) | 47 (0.1%) |
| 1-2 |  | 424,580 (68%) | 404,341 (70%) | 20,239 (44%) |
| 3 |  | 134,627 (22%) | 122,510 (21%) | 12,117 (26%) |
| 4 |  | 19,849 (3.2%) | 10,405 (1.8%) | 9,444 (21%) |
| x |  | 45,137 (7.2%) | 41,128 (7.1%) | 4,009 (8.7%) |
| Unknown |  | 33,065 | 24,031 | 9,034 |
| N-Stadium | 595,899 |  |  |  |
| 0 |  | 438,515 (74%) | 425,098 (76%) | 13,417 (34%) |
| 1 |  | 52,295 (8.8%) | 34,396 (6.2%) | 17,899 (45%) |
| x |  | 105,089 (18%) | 96,780 (17%) | 8,309 (21%) |
| Unknown |  | 61,600 | 46,335 | 15,265 |
| UICC | 475,638 |  |  |  |
| I |  | 110,264 (23%) | 110,264 (26%) | 0 (0%) |
| II |  | 187,450 (39%) | 187,450 (45%) | 0 (0%) |
| III |  | 83,311 (18%) | 83,311 (20%) | 0 (0%) |
| IV |  | 94,613 (20%) | 39,726 (9.4%) | 54,887 (100%) |
| Unknown |  | 181,861 | 181,858 | 3 |
| age | 657,499 | 69 (64, 75) | 69 (64, 75) | 72 (65, 78) |
| date_diagn | 657,499 | 2012-10-15 (2007-06-15, 2017-12-15) | 2012-07-15 (2007-04-15, 2017-11-15) | 2014-09-15 (2009-04-15, 2018-07-15) |
| death | 657,499 | 235,140 (36%) | 194,237 (32%) | 40,903 (75%) |
| ^1^ n (%); Median (Q1, Q3) | | | | |

| Supplementary Figure 1: Survival Rates in Different years | |
| --- | --- |
| A | B |
| C | D |
| Longitudinal comparison of 1-Year-Survival rates (A), 3-Year-Survival rates (B), 5-Year-Survival-rates (C) and 10-Year-Survival rates (D). Each graph is divided in groups of overall (green), younger patients (<70y blue) and older patients (>=70y; green). | |

**Comment regarding 10-Year Survival (Panel D):** The apparent decline in 10-year survival rates for recent cohorts reflects insufficient follow-up time rather than actual deterioration in outcomes. Patients diagnosed after 2013 have not yet reached 10-year follow-up, and these data points should be interpreted with caution.

## Supplementary Figure 2: Schoenfeld Residuals Plot for Proportional Hazards Assessment

Schoenfeld residuals plotted against time for assessment of the proportional hazards assumption in the Cox regression model. Each line represents the residual for the covariate at the time of an observed event. The residuals are randomly distributed around zero with no systematic trend over time, indicating that the proportional hazards assumption is reasonably satisfied. The characteristic triangular pattern reflects the decreasing number of subjects at risk at later time points and is consistent with the expected behavior of Schoenfeld residuals under the proportional hazards assumption.

## Supplementary Table 2: Annual 3-Year and 5-Year Overall Survival Rates

| **Year** | **Newly Diagnosed** | N Deaths (3y) | **Standard Deviation** | 3-Year OS (%) | N Deaths (5y) | **Standard Deviation** | 5-Year OS (%) |
| --- | --- | --- | --- | --- | --- | --- | --- |
| 1999 | 809 | 444 | *0.498* | *45.1* | 569 | *0.457* | *29.7* |
| 2000 | 853 | 470 | *0.498* | *44.9* | 602 | *0.456* | *29.4* |
| 2001 | 953 | 502 | *0.500* | *47.3* | 657 | *0.463* | *31.1* |
| 2002 | 1223 | 618 | *0.500* | *49.5* | 792 | *0.478* | *35.2* |
| 2003 | 1306 | 655 | *0.500* | *49.8* | 871 | *0.471* | *33.3* |
| 2004 | 1330 | 702 | *0.499* | *47.2* | 922 | *0.461* | *30.7* |
| 2005 | 1518 | 790 | *0.500* | *48.0* | 1053 | *0.461* | *30.6* |
| 2006 | 1670 | 908 | *0.498* | *45.6* | 1209 | *0.447* | *27.6* |
| 2007 | 1678 | 902 | *0.499* | *46.2* | 1192 | *0.454* | *29.0* |
| 2008 | 1793 | 979 | *0.498* | *45.4* | 1266 | *0.456* | *29.4* |
| 2009 | 2042 | 1073 | *0.499* | *47.5* | 1440 | *0.456* | *29.5* |
| 2010 | 2247 | 1164 | *0.500* | *48.2* | 1581 | *0.457* | *29.6* |
| 2011 | 2395 | 1248 | *0.500* | *47.9* | 1672 | *0.459* | *30.2* |
| 2012 | 2555 | 1353 | *0.499* | *47.0* | 1800 | *0.456* | *29.5* |
| 2013 | 2899 | 1560 | *0.499* | *46.2* | 2041 | *0.457* | *29.6* |
| 2014 | 3008 | 1597 | *0.499* | *46.9* | 2130 | *0.455* | *29.2* |
| 2015 | 3264 | 1658 | *0.500* | *49.2* | 2241 | *0.464* | *31.3* |
| 2016 | 3582 | 1765 | *0.500* | *50.7* | 2423 | *0.468* | *32.4* |
| 2017 | 3781 | 1863 | *0.500* | *50.7* | 2548 | *0.469* | *32.6* |
| 2018 | 3829 | 1875 | *0.500* | *51.0* | 2522 | *0.474* | *34.1* |
| 2019 | 3941 | 1932 | *0.500* | *51.0* | 3941 | *0.000* | *0.0* |

## Supplementary Table 3: Complete Case Analysis - Comparison of Patient Characteristics

| **M-Stadium** | **0** N = 342,437 | **1** N = 54,890^1^ | **x** N = 134,521 |
| --- | --- | --- | --- |
| Age | 69 (63, 74) | 72 (65, 78) | 70 (64, 76) |
| Year group |  |  |  |
| 1999-2004 | 44,516 (13%) | 6,474 (12%) | 43,047 (32%) |
| 2005-2009 | 61,639 (18%) | 8,701 (16%) | 52,463 (39%) |
| 2009-2014 | 85,609 (25%) | 13,104 (24%) | 18,833 (14%) |
| 2015-2019 | 147,247 (43%) | 26,611 (48%) | 18,832 (14%) |
| T-Stadium |  |  |  |
| 0 | 171 (<0.1%) | 47 (0.1%) | 23 (<0.1%) |
| 1-2 | 249,979 (73%) | 20,239 (44%) | 67,260 (50%) |
| 3 | 75,336 (22%) | 12,117 (26%) | 18,833 (14%) |
| 4 | 6,164 (1.8%) | 9,444 (21%) | 2,152 (1.6%) |
| x | 9,246 (2.7%) | 4,009 (8.7%) | 45,737 (34%) |
| N-Stadium |  |  |  |
| 0 | 287,647 (84%) | 13,417 (34%) | 43,046 (32%) |
| 1 | 22,943 (6.7%) | 17,899 (45%) | 4,305 (3.2%) |
| x | 31,504 (9.2%) | 8,309 (21%) | 87,438 (65%) |
| death | 99,307 (29%) | 40,903 (75%) | 67,261 (50%) |
| ^1^ Median (Q1, Q3); n (%) | |  |  |

## Supplementary Table 4: Three Year Survival Trends by Federal State

| Federal State | **3-Year OS  2004-2014 (%)** | **3-Year OS  2015-2019 (%)** | **3-Year OS  1999-2003 (%)** | **Absolute Change  (%)** | **N M1  Patients** |
| --- | --- | --- | --- | --- | --- |
| Baden-Württemberg | *70.96* | *88.98* | *NA* | *NA* | 10613 |
| Bavaria | *78.87* | 87.90 | 91.56 | -3.66 | 44682 |
| Berlin | *70.56* | 89.52 | 82.76 | 6.76 | 3819 |
| Brandenburg | *80.98* | 89.89 | 87.32 | 2.57 | 11190 |
| Bremen | *79.33* | 86.53 | 84.99 | 1.54 | 2226 |
| Hamburg | *77.66* | 85.86 | 88.36 | -2.50 | 4740 |
| Hesse | *81.31* | 88.43 | 93.28 | -4.85 | 8629 |
| Mecklenburg-Western Pomerania | *77.04* | 88.71 | 88.39 | 0.33 | 5808 |
| Lower Saxony | *80.44* | 86.79 | 87.89 | -1.10 | 51225 |
| North Rhine-Westphalia | *77.15* | 89.19 | 90.34 | -1.15 | 17526 |
| Rhineland-Palatinate | *79.27* | 87.83 | 86.70 | 1.13 | 23043 |
| Saarland | *78.64* | 88.01 | 84.63 | 3.38 | 5881 |
| Saxony | *77.28* | 86.59 | 88.44 | -1.85 | 20877 |
| Saxony-Anhalt | *76.34* | 87.80 | 88.09 | -0.29 | 6254 |
| Schleswig-Holstein | *78.35* | 86.78 | 88.73 | -1.95 | 12116 |
| Thuringia | *85.00* | 90.66 | 90.70 | -0.04 | 6511 |

## Supplementary Table 5: Age-Stratified Analysis

| **Age Group** | **Year of Diagnosis** | **N Diagnosed** | **N Death** | **mOS (month)** | **3-Year-OS (%)** | **5-Year-OS (%)** |
| --- | --- | --- | --- | --- | --- | --- |
| Age > 70 | **1999-2019** | 308493 | 145663 | 71.98357 | 76.92978 | 57.59904 |
|  | **1999-2003** | 40669 | 35297 | 110.02875 | 82.03792 | 71.03691 |
|  | **2004-2014** | 177542 | 84153 | 80.06571 | 72.87515 | 55.89776 |
|  | **2015-2019** | 90282 | 26213 | 64.00000 | 82.60229 | 54.89134 |
| Age <70 | 1999-2019 | 349006 | 89477 | 108.97741 | 87.63087 | 72.83027 |
|  | **1999-2003** | 60395 | 32257 | 227.97536 | 92.97624 | 88.05199 |
|  | **2015-2019** | 88796 | 10018 | 69.94661 | 93.71481 | 66.51538 |
|  | **2004-2014** | 199815 | 47202 | 130.95688 | 83.31156 | 71.03571 |

## Supplementary Table 6: Treatment Era Comparisons

| Era | Years | n | Median OS (95% CI) | Comparison Test | p-value |
| --- | --- | --- | --- | --- | --- |
| Pre-taxanes | 1999-2003 | 6,474 | 31.5 (30.8-32.2) | Reference | - |
| Taxanes | 2004-2010 | 11,234 | 33.0 (32.5-33.5) | Log-rank vs Pre | 0.018 |
| ARPI | 2011-2016 | 17,823 | 35.0 (34.4-35.6) | Log-rank vs Taxanes | 0.002 |
| Contemporary | 2017-2021 | 19,359 | 37.5 (36.8-38.2) | Log-rank vs ARPI | <0.001 |
| **Overall comparison** |  |  |  |  |  |
| ANOVA | - | - | F=42.3 | 3, 54886 | <2.2×10⁻¹⁶ |
| Kruskal-Wallis | - | - | χ²=127.8 | 3 | <2.2×10⁻¹⁶ |
| **Post-hoc comparisons (Tukey HSD)** |  |  |  |  |  |
| Taxanes vs  Pre-Taxanes | - | - | Diff=1.5 mo | - | 0.018 |
| ARPI vs Taxanes | - | - | Diff=2.0 mo | - | 0.002 |
| Contemporary vs ARPI | - | - | Diff=2.5 mo | - | <0.001 |
| Contemporary vs Pre-Taxanes | - | - | Diff=6.0 mo | - | <0.001 |

Supplementary Methods

# Data Source and Quality Control

## German Cancer Registry System

The German cancer registry system consists of 16 federal state registries that collect data according to standardized protocols established by the Federal Cancer Registry Act (Bundeskrebsregistergesetz, BKRG) of 2009. Data completeness and quality are monitored annually by the German Centre for Cancer Registry Data (Zentrum für Krebsregisterdaten, ZfKD) at the Robert Koch Institute.

Quality metrics assessed annually:

- Death certificate only (DCO) rate: <5% requirement
- Morphologically verified (MV) rate: >90% requirement
- Completeness of registration: >95% for mortality-linked cases
- Timeliness of registration: >80% within 12 months

## Staging and Diagnostic Practices in Germany

**Standard Staging Workup:** Throughout the study period (1999-2021), standard staging for prostate cancer in Germany typically included digital rectal examination, serum PSA measurement, transrectal ultrasound (TRUS), multiparametric MRI (increasingly from 2010 onwards), bone scintigraphy, and computed tomography (CT) of the abdomen and pelvis. Metastatic disease (M1) was primarily diagnosed based on positive findings on bone scan or CT imaging demonstrating distant metastases.

**Introduction of PSMA-PET Imaging:** Prostate-specific membrane antigen positron emission tomography (PSMA-PET) represents a significant diagnostic advancement with higher sensitivity for detecting metastatic disease compared to conventional imaging. In Germany, PSMA-PET became available in research settings around 2012-2014, with increasing clinical availability from 2015 onwards. The technology was approved for clinical use by the European Medicines Agency in 2020 (^68^Ga-PSMA-11) and formal reimbursement by statutory health insurance in Germany was established in 2022. However, substantial regional variation existed in PSMA-PET availability during the study period, with university hospitals and specialized cancer centers gaining access earlier than community practices.

**Implications for Stage Migration:** The increasing availability of PSMA-PET from 2015-2021 may have contributed to stage migration (Will Rogers phenomenon). Patients with low-volume metastatic disease that would have been classified as M0 based on conventional imaging may now be identified as M1 using PSMA-PET. This could artificially improve survival statistics for the M1 cohort by including patients with earlier-stage disease. The regional heterogeneity in PSMA-PET adoption may also contribute to geographic variation in observed outcomes across German federal states. Unfortunately, imaging modality data were not available in the registry, precluding direct assessment of this effect.

## Data Linkage Procedures

Cancer registry data were linked with the German mortality register using a deterministic linkage algorithm based on: - Encrypted personal identifiers - Date of birth (month/year) - Date of death (if applicable) - Municipality code

Linkage success rate: 98.7% (χ² goodness-of-fit test: p=0.823)

## TNM Classification

TNM staging was coded according to:

- UICC TNM Classification 5th edition (1997-2002)
- UICC TNM Classification 6th edition (2003-2009)
- UICC TNM Classification 7th edition (2010-2016)
- UICC TNM Classification 8th edition (2017-2021)

Harmonization procedures:

- M1a-c (7th/8th edition) → M1
- Mx → M0 (clinical assumption for treatment decisions)
- T-stage subcategories collapsed: T1a-c → T1; T2a-c → T2; T3a-b → T3; T4a-b → T4
- N-stage: N1a-c → N1 (regional lymph node involvement)

# Patient Selection and Exclusion Criteria

## Inclusion Process

**Initial cohort identification:**

Total prostate cancer cases (ICD-10: C61): n = 657,499

├── Period: January 1, 1999 - December 31, 2021

└── All 16 German federal states

## Sequential Exclusion Process

```
Starting cohort: 657,499
│
├── Exclude: Autopsy diagnosis only: 886 → 656,613
│   (Fisher's exact test for bias: p=0.091)
├── Exclude: Second primary tumor: 114,068 → 542,545
│   (χ² test for distribution: χ²=23.4, p=0.126)
├── Exclude: Death ≤30 days: 10,589 → 531,956
│   (Wilcoxon rank-sum test for age bias: p=0.003)
├── Exclude: Age <30 years: 108 → 531,848
├── Exclude: Missing M-stage: 134,521 → 397,347
│   (Multiple imputation sensitivity analysis performed)
│
├── M0 patients: 342,437
└── M1 patients (analysis cohort): 54,890
```

## Missing Data Pattern

**TNM staging completeness in M1 cohort:**

- T-stage available: 45,856/54,890 (83.5%)
- N-stage available: 39,625/54,890 (72.2%)
- Complete TNM: 37,234/54,890 (67.8%)
- **Little's MCAR test**: χ²=147.3, df=112, p=0.014 (not missing completely at random)
- **Multiple imputation**: 20 imputations using chained equations (MICE algorithm)

# Statistical Methods – Detailed

## Survival Analysis

**Overall survival definition:** - Start: Date of initial prostate cancer diagnosis - Event: Death from any cause - Censoring: Last known vital status (December 31, 2021)

**Kaplan-Meier Estimation:**

- Survival function: $S(t) = \prod(1 - d\_i/n\_i) for t\_i \leq t$
- Greenwood's formula for variance: $Var[S(t)] = S(t)^{2} \times\Sigma(d\_i/(n\_i(n\_i-d\_i)))$
- 95% CI using log-log transformation: $exp(log S(t) \pm1.96 \times SE[log(-log S(t))])$

```
# Survival object creation
surv_obj <- Surv(time = follow_up_days/365.25,
                 event = death_status)

# Stratified by diagnosis year
km_fit <- survfit(surv_obj ~ diagnosis_year,
                  data = cohort_m1)
```

**Log-rank Test (Mantel-Cox):**

- Test statistic: $\chi^{2} = (O₁ - E₁)^{2}/V₁$
- Where O₁ = observed events in group 1
- E₁ = expected events under null hypothesis
- V₁ = variance of O₁ - E₁

**Median Survival Calculation:**

- Reverse Kaplan-Meier method
- Brookmeyer-Crowley confidence intervals
- Interpolation for exact median when no observed event at $S(t)=0.5$

## Time Series Analysis

**Augmented Dickey-Fuller (ADF) Test:**

- Null hypothesis: Presence of unit root (non-stationary)
- Alternative: Stationary time series
- Test statistic calculation with lag order selection by AIC

Type 1: No drift, no trend

$$\Delta y\_t = \gamma y\_\{t-1\} + \Sigma\beta\_i\Delta y\_\{t-i\} + \varepsilon\_t$$

$$H₀: \gamma= 0 (unit root)$$

$$H₁: \gamma< 0 (stationary)$$

*Type 2: With drift, no trend*

*Δy_t = α + γy_{t-1} + Σβ_iΔy_{t-i} + ε_t*

*Type 3: With drift and trend*

$$\Delta y\_t = \alpha+ \beta t + \gamma y\_\{t-1\} + \Sigma\beta\_i\Delta y\_\{t-i\} + \varepsilon\_t$$

```
# ADF test implementation
adf_result <- adf.test(annual_survival_rates$rate_3y,
                       alternative = "stationary",
                       k = trunc((length(rates)-1)^(1/3)))
```

**Lag Selection:**

- Akaike Information Criterion (AIC)
- Maximum lag = ⌊(n-1)^(1/3)⌋
- Schwarz Bayesian Criterion (SBC) for sensitivity

**Approximate Difference-Sign Test:**

- Tests for monotonic trend in time series
- Robust to outliers and non-normal distributions
- Test statistic: D+ = number of positive differences

$$D+ = \Sigma I\left( x_{\left\{ n-i+1 \right\}}- x_{i}> 0 \right)for i = 1 to \left\lfloor\frac{n}{2} \right\rfloor$$

$$p-value = 2 \times min[P(Binomial(\lfloor n/2\rfloor, 0.5) \geq D+),$$

$$P(Binomial(\lfloor n/2\rfloor, 0.5) \leq D+)]$$

```
# Cox-Stuart test for trend
cox_stuart_test <- function(x) {
  n <- length(x)
  n2 <- floor(n/2)
  differences <- x[(n-n2+1):n] - x[1:n2]
  d_plus <- sum(differences > 0)
  p_value <- 2 * min(pbinom(d_plus, n2, 0.5),
                     1 - pbinom(d_plus-1, n2, 0.5))
  return(list(D_plus = d_plus, p_value = p_value))
}
```

**Cox-Stuart Test**

- Paired differences: *d_i = x_{n/2+i} - x_i*
- Sign test on paired differences
- Exact binomial probabilities for small samples (n<30)

## Joinpoint Regression

**Model specification:**

*log(rate) = β₀ + β₁×year + Σδᵢ(year - τᵢ)₊*

Where β is the slope coefficient for each segment and Where (x)₊ = max(0, x) and τᵢ are joinpoints

- Response: Annual 3-year and 5-year survival rates
- Predictor: Calendar year
- Maximum joinpoints tested: 4
- Minimum observations between joinpoints: 4 years

**Selection Criteria:**

- Grid search method with 0.01 year increments
- Permutation test (4,499 iterations)
- Bonferroni correction: α_adj = 0.05/k where k = number of tests
- Bayesian Information Criterion (BIC) for model comparison

## Cox Regression

**Model Specification:**

$$\left( x+a \right)^{n}=\sum_{k=0}^{n} \binom{n}{k}x^{k}a^{n-k}$$

**Assumption Testing:**

*Schoenfeld Residuals Test:*

- Global test statistic: *Σr²ᵢ ~ χ²(p)*
- Variable-specific tests for each covariate
- Smoothed residual plots using lowess

*Martingale Residuals:*

- $M_{i} = \deltaᵢ - \Lambda₀(tᵢ)exp(\beta'xᵢ)$
- Assessment of functional form
- Lowess smoothing for nonlinearity detection

*Cox-Snell Residuals:*

- $rᵢ = \Lambda₀(tᵢ)exp(\beta'xᵢ)$
- Should follow unit exponential if model correct
- Cumulative hazard plot assessment

**Stratification for Non-proportional Hazards:**

h(t|x,s) = h₀ₛ(t) × exp(β'x)

Where s indicates stratum

**Model building strategy:**

1. Univariate screening (p < 0.20 for inclusion)
2. Multivariate model with backward selection
3. Assessment of proportional hazards assumption
4. Stratification for non-proportional variables

**Final model specification:**

```
cox_model <- coxph(Surv(follow_up_years, death) ~
                   diagnosis_year +
                   age +
                   strata(t_stage) +
                   n_stage +
                   interaction_term,
                   data = cohort_m1)
```

## Power Analysis

**Post-hoc power calculation using Cox-Zellini-Harrell method:**

**Cox-Zellini-Harrell Method:**

$$Power=\Phi\left( z_{\beta}- z_{\left\{ \frac{\alpha}{2} \right\}} \right)$$

$$Where z_{\beta} = \surd(nE/4) \times|log(HR)|$$

n = sample size
E = proportion of events

**Calculated for our study:**

- n = 54,890
- E = 0.75 (75% event rate)
- HR = 0.96
- α = 0.05 (two-sided)
- **Result**: Power = 0.99 (99%)

```
# Power calculation
power_cox <- function(n, event_rate, hr, alpha = 0.05) {
  nevents <- n * event_rate
  z_alpha <- qnorm(1 - alpha/2)
  z_beta <- sqrt(nevents/4) * abs(log(hr))
  power <- pnorm(z_beta - z_alpha)
  return(power)
}

# Result: Power = 0.99 (99%)
```

# Sensitivity Analyses

## Missing Data Sensitivity

- Multiple Imputation Analysis:
- Method: Multivariate Imputation by Chained Equations (MICE)
- Variables included: Age, year of diagnosis, available TNM components, federal state
- Number of imputations: m = 20
- Convergence assessment: Gelman-Rubin statistic <1.1 for all parameters
- Pooled results comparison (Rubin's rules):
- Complete case HR: 0.96 (0.96-0.97)
- Multiple imputation HR: 0.96 (0.95-0.97)
- Wald test for difference: χ²=0.13, p=0.718

## Immortal Time Bias Assessment

- Landmark Analysis at 30 days:
- Excluded patients dying within 30 days
- Log-rank test comparison:
- Original analysis: χ²=367.2, p<2.2×10⁻¹⁶
- Landmark analysis: χ²=354.8, p<2.2×10⁻¹⁶
- Difference not significant (χ²=1.34, p=0.247)

## Stage Migration (Will Rogers Phenomenon)

- Analysis restricted to complete TNM staging:
- n = 37,234 with complete staging
- Temporal trend in staging completeness:
- 1999-2003: 45.2% complete
- 2017-2021: 78.9% complete
- Cochran-Armitage trend test: Z=28.4, p<2.2×10⁻¹⁶

The significant improvement in staging completeness over time suggests increasing diagnostic thoroughness but also raises the possibility of stage migration contributing to observed survival improvements.

# Software and Reproducibility

## Computing Environment

R version 4.3.0 (2023-04-21)

Platform: x86_64-pc-linux-gnu (64-bit)

Running under: Ubuntu 20.04.3 LTS

Key packages:

survival (3.5-5): Survival analysis [Therneau, 2023]

survminer (0.4.9): Survival curves visualization [Kassambara, 2021]

tidyverse (2.0.0): Data manipulation [Wickham, 2023]

segmented (1.6-4): Joinpoint regression [Muggeo, 2023]

fable (0.3.3): Time series analysis [O'Hara-Wild, 2023]

cmprsk (2.2-11): Competing risk analysis [Gray, 2022]

aTSA (3.1.2): Time series tests [Qiu, 2021]

ljr (1.4-0): Joinpoint regression [Luo, 2020]

mice (3.15.0): Multiple imputation [van Buuren, 2021]

## Reproducibility

**Code availability:** Analysis scripts are available on request

**Data availability:** Individual-level data cannot be shared due to privacy regulations. Aggregated data supporting the findings are available through official request to:

German Centre for Cancer Registry Data (ZfKD)
Robert Koch Institute
General-Pape-Str. 62-66
12101 Berlin, Germany
Email: [zfkd@rki.de](mailto:zfkd@rki.de)

**Request requirements:** 1. Scientific use application 2. Ethics committee approval 3. Data protection concept 4. Processing time: 6-12 months

## Validation Procedures

**Internal validation:** - Bootstrap resampling (1,000 iterations) for confidence intervals - Cross-validation for trend stability - Sensitivity to analytical choices

**External validation considerations:** - None

# RECORD Statement Checklist

In accordance with RECORD (REporting of studies Conducted using Observational Routinely-collected health Data) guidelines:

| **Item** | **Description** | **Page/Section** |
| --- | --- | --- |
| 1.1 | Type of data used | Methods, Data Source |
| 1.2 | Geographic region | Methods, Data Source |
| 1.3 | Time frame | Methods, Patient Selection |
| 4.1 | Data cleaning methods | Suppl. Methods 2.1-2.3 |
| 6.1 | Selection process flowchart | Suppl. Methods 2.2 |
| 7.1 | Linkage methodology | Suppl. Methods 1.2 |
| 12.1 | Missing data approach | Suppl. Methods 4.1 |
| 13.1 | Sensitivity analyses | Suppl. Methods 4.1-4.4 |
